# Integration of gene expression and DNA methylation data using MLA-GNN for liver cancer biomarker mining

**DOI:** 10.3389/fgene.2024.1513938

**Published:** 2024-12-23

**Authors:** Chun-Yu Lu, Zi Liu, Muhammad Arif, Tanvir Alam, Wang-Ren Qiu

**Affiliations:** ^1^ School of information engineering, Jingdezhen Ceramic University, Jingdezhen, China; ^2^ College of Science and Engineering, Hamad Bin Khalifa University, Doha, Qatar

**Keywords:** multi-omics data, feature selection, MLA-GNN, Cartesian product, WGCNA

## Abstract

The early symptoms of hepatocellular carcinoma patients are often subtle and easily overlooked. By the time patients exhibit noticeable symptoms, the disease has typically progressed to middle or late stages, missing optimal treatment opportunities. Therefore, discovering biomarkers is essential for elucidating their functions for the early diagnosis and prevention. In practical research, challenges such as high-dimensional features, low sample size, and the complexity of gene interactions impact the reliability of biomarker discovery and disease diagnosis when using single-omics approaches. To address these challenges, we thus propose, Multi-level attention graph neural network (MLA-GNN) model for analyzing integrated multi-omics data related to liver cancer. The proposed protocol are using feature selection strategy by removing the noise and redundant information from gene expression and DNA methylation data. Additionally, it employs the Cartesian product method to integrate multi-omics datasets. The study also analyzes gene interactions using WGCNA and identifies potential genes through the MLA-GNN model, offering innovative approaches to resolve these issues. Furthermore, this paper identifies FOXL2 as a promising liver cancer marker through gene ontology and survival analysis. Validation using box plots showed that the expression of the gene FOXL2 was higher in patients with hepatocellular carcinoma than in normal individuals. The drug sensitivity correlation and molecular docking results of FOXL2 with the liver cancer-targeting agent lenvatinib emphasized its potential role in hepatocellular carcinoma treatment and highlighted the importance of FOXL2 in hepatocellular carcinoma treatment.

## 1 Introduction

Liver hepatocellular carcinoma (LIHC) is a globally prevalent cancer with increasing incidence and mortality in recent years (Sevic et al., 2019). In China, the annual incidence of LIHC ranks fifth among all types of cancer, and its mortality rate even exceeds that of the second-ranked one (Acharya and Mukhopadhyay, 2024). Besides, LIHC is the only cancer with annually increasing incidence (Sun et al., 2020).

With the popularization of high-throughput technologies, artificial intelligence and multi-omics data are increasingly utilized in tumor therapy research. Cancer-related biomarkers can be more accurately identified by using multi-omics data (Kanchan et al., 2024). Integrating data across gene mutations, protein expression, and metabolite levels offers a robust foundation for cancer diagnosis and prognosis (Acharya and Mukhopadhyay, 2024). However, the traditional assumption of independent and identically distributed may not universally apply to gene expression (GE) (Palsson and Zengler, 2010). Advanced methodologies are required to assess gene interactions and correlations effectively. Integrating and analyzing multi-omics data presents a complex challenge due to variations and interrelationships across different data types. Moreover, the high-dimension disaster and low-sample size (HDLSS) features is still challenging to address.

In this paper, we utilized the Cartesian product to integrate gene expression and DNA methylation datasets of LIHC and MLA-GNN model for n-depth analysis of integrated multi-omics data and its implications for cancer research. For instance, previous studies have successfully applied multi-omics data for predicting Alzheimer’s disease, demonstrating the utility of the Cartesian approach (Park et al., 2020). In addition, MLA-GNN has been used to explore gene modules and topological information in histology data (Xing et al., 2022). The association between potential geneses and LIHC were further investigated through survival analysis, gene ontology analysis, literature review and drug correlation analysis, and found that FOXL2 may be associated with LIHC as potential biomarkers.

### 1.1 Related work

Advances in sequencing technology have led to the rapid accumulation of extensive cancer genome data. However, the curse of dimensionality and redundancy in the cancer gene data is significantly higher than the number of available instances (Zhang et al., 2011). HDLSS issues pose significant challenges in bioinformatics and medical research, making dimensionality reduction an essential technique for managing such data (Tang, 2020).

In the dimensionality reduction algorithm, feature selection methods aim to choose the most representative subset of features from the original dataset, creating a more explanatory set. This approach is highly effective in high-dimensional data like GE, where selected features hold significant biological relevance (Afshar and Usefi, 2020). For example, the “MI”(Tang and Zhou, 2016) and SNR (Hamraz et al., 2023) are utilized for feature selection to enhance classification accuracy.

Multi-omics data enables a comprehensive analysis of the entire genome, promising significant advancements in genetic data precision and disease prediction reliability. Moreover, recent studies increasingly employ Machine Learning techniques such as Random Forest (Breiman, 2001), Support Vector Machine (Huang et al., 2018) and Neural Networks (Picard et al., 2021) to analyze histological data, achieving notable success in Gene Expression classification and prediction.

The use of graph neural networks has advanced histological analysis significantly (Wang et al., 2021). For instance, one study employed a curated database to build a gene graph and utilized a deep feed-forward network embedded within the graph to predict disease outcomes (Wysocki and Ritter, 2011). These approaches not only enhance classification accuracy but also offer more interpretable biomarkers.

## 2 Methods

**FIGURE 1:**
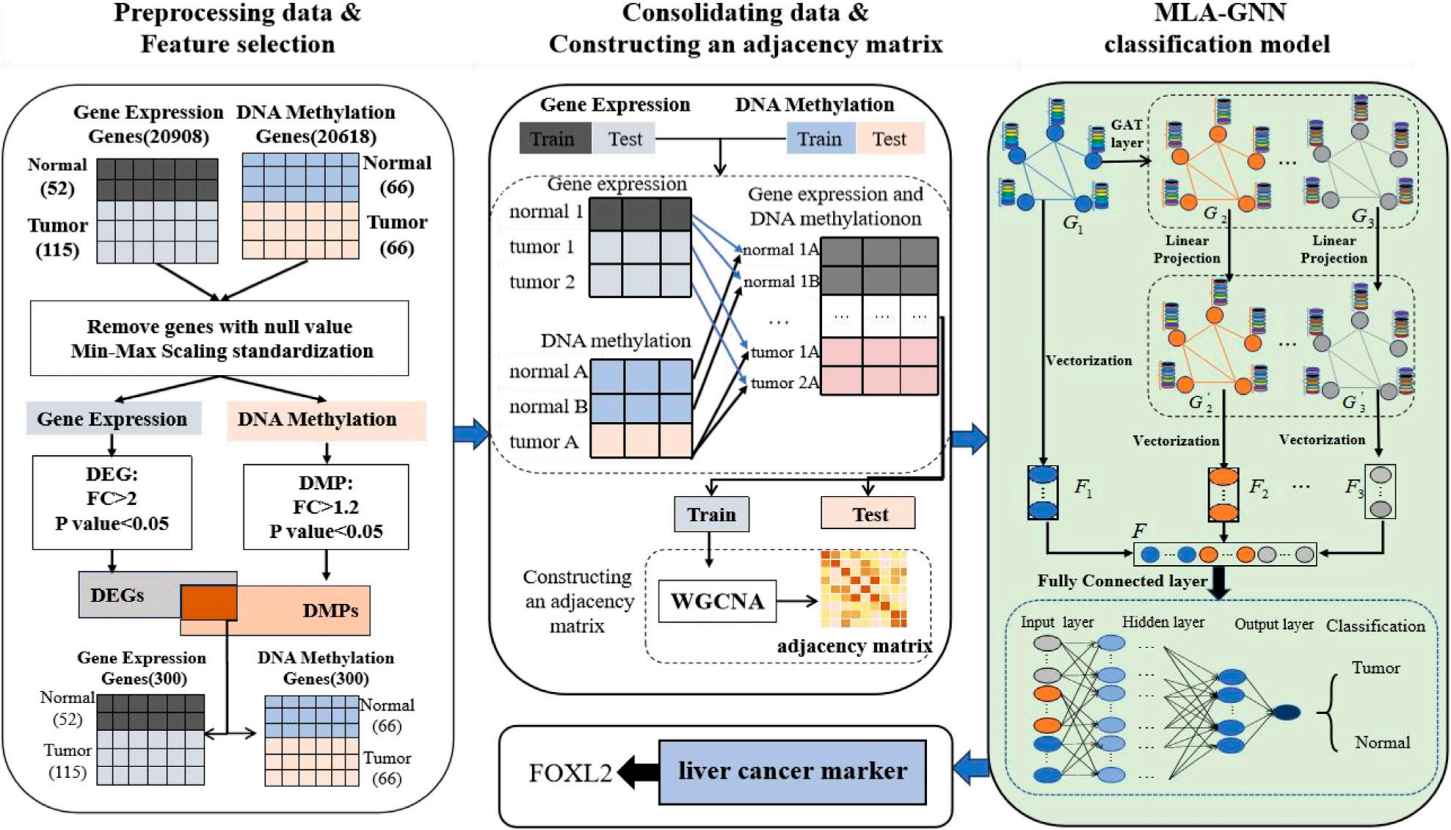
Workflow of hepatocellular carcinoma prediction and biomarker recognition.

In this section, we provide an overview of the LIHC-based biomarker discovery process. This process comprises three main stages: data preprocessing and feature selection, data combination and constructioning an adjacency matrix, and MLA-GNN (Figure 1).

### 2.1 Data collection and preprocessing

The relevant GE dataset (GSE76427) and DNA methylation dataset (GSE54503) were obtained and analyzed using the GEO database platform (https://www.ncbi.nlm.nih.gov/geo/) (Davis and Meltzer, 2007). Additionally, clinical data were integrated and analyzed to gain a deeper understanding of the role of these genes in the immune system. These clinical data were sourced from The Cancer Genome Atlas (TCGA, https://portal.gdc.cancer.gov/), which offers extensive clinical information on cancer patients.

In the data preprocessing phase, the raw data underwent background correction, normalization, and genotype re-annotation. These critical steps were carried out using the “limma” package in R software to eliminate experimental noise and enhance data quality and comparability (Ramírez-Gallego et al., 2017).

According to the platform annotation file, map the probes in the CEL files of GE and DNA methylation data to the corresponding genes. If multiple probes correspond to the same gene, their average is taken and removed the genes with missing values.

In the preprocessing of the DNA methylation dataset, after cleaning and transformation, we obtained a dataset containing 20,908 genes. Duplicated genes were merged by averaging their values within each group (patients and normals), and missing values were imputed with the mean of their respective group. A similar cleaning and transformation process was applied to the gene expression data. This resulted in a dataset of 20,606 genes, which will be used for subsequent differential gene analysis and functional studies (Table 1).

**TABLE 1:** Dataset summary.

Dataset	GE	DNA methylation
GEO ID	GSE76427	GSE54503
Normal samples	115	66
LIHC samples	51	66
Genes	20,908	20,606
upregulated gene	9,932	10,839
Common upregulated genes	300 (DEGs: FC > 2, P value < 0.05) (DMPs: FC > 1. 2, P value < 0.05)	

The two datasets were normalized in order to avoid the effects of integrating the two dataset gauges. The Min-Max Normalization method was employed in this study to ensure uniformity across all data, facilitating reliable comparisons between the different datasets.

To assess the model’s performance, the dataset was randomly split into training and test sets in a 7: 3 ratio. The training set was utilized to build and train the model, whereas the test set was employed to validate the model’s predictive capabilities.

### 2.2 Differential gene identification

After data preprocessing, volcano plots, PCA, and heat maps were used to demonstrate whether the preprocessed gene expression and DNA methylation data as a whole clearly differentiated patients from normal individuals.

**FIGURE 2:**
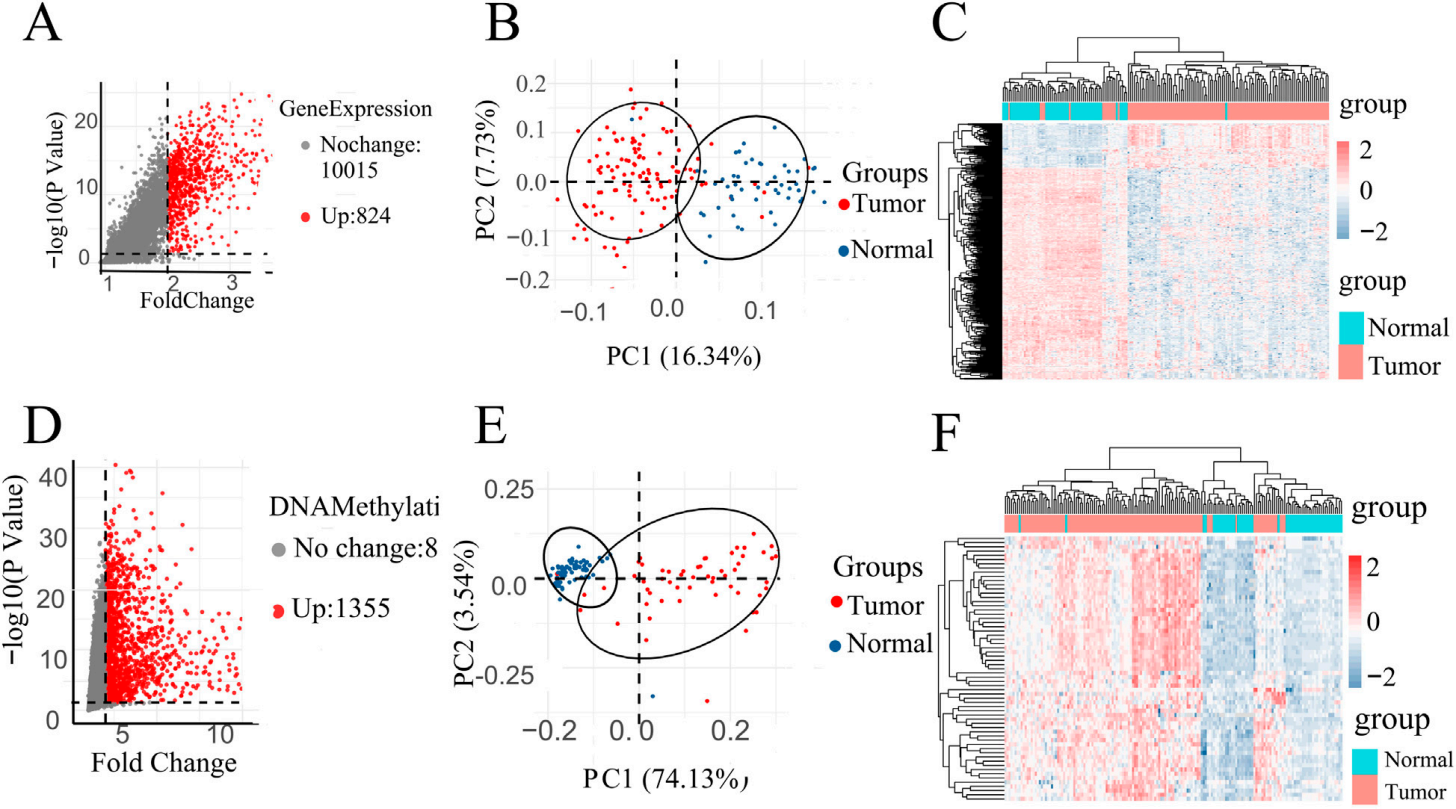
**(A)** Volcano map of DEGs in GE data. **(D)** Volcano map of DMPs in DNA methylation data. PCA of all instances in **(B)** GSE67427 and **(E)** GSE54503 dataset represent two distinct groups. All instances Heatmap in **(C)** GSE67427 and **(F)** GSE54503.

A volcano plot is a type of scatterplot that illustrates statistical significance (p-value) against the magnitude of change (fold change). depict volcano plots based on GE data and DNA methylation data, correspondingly. On the plot, each point denotes an identified gene: the upregulated genes are depicted by the red points, while gray points denote genes without significant differences (Figures 2A, D). Vertical black lines indicate genes with a fold change (FC) greater than 2, and horizontal lines mark genes with p-values less than 0.05, indicating significant differential expression. Smaller p-values indicate greater significance in gene expression differences.

Specifically, highlights 824 upregulated genes in the DNA methylation dataset under specific conditions, while focuses on 1,355 upregulated genes in gene expression (Figures 2A, D).

In order to investigate the data plausibility, principal component analysis (PCA) and heat maps were conducted. PCA plots (Figures 2B, E) for GSE54503 and GSE76427 were used to visualize sample distribution. Points in the PCA plot represent samples, with greater distances between points indicating larger differences between samples. Both plots show distinct clustering of normal and tumor samples, suggesting significant differences between LIHC samples and normal samples with a well-defined data distribution.

In the heat map (Figures 2C, F), the horizontal axes represent normal and patient samples. The upper part of the graph uses blue to denote normal samples and red to denote patient samples. Utilizing K-means clustering, the heat map employs upregulated genes from the volcano map as horizontal coordinates, demonstrating complete differentiation between patients and normal individuals. These results underscore the meaningful selection and analysis of data.

The screening of differential genes for gene expression and DNA methylation based on volcano plots, as well as the PCA plots and heat maps of the screened differential genes, respectively, showed that they all clearly differentiated patients from normal people, suggesting that the gene expression and DNA methylation datasets are biologically significant in distinguishing between the patient and normal populations.

### 2.3 Feature selection method

Despite data preprocessing, challenges persisted with HDLSS, which increased the risk of overfitting and gradient variance. To address these issues, a biomarker selection method was employed. Specifically, the Fold Change (FC) was utilized. This method calculates the ratio of average gene expression levels between two sample groups. Genes surpassing a predefined threshold are identified as differentially expressed. Additionally, the t-test was applied to compute a t-statistic for each gene to quantify expression differences between sample types. The resulting p-value, derived from the t-distribution, assesses the significance of these differences

We intersected genes based on biometric feature selection criteria (Table 1). This intersection targeted genes that were differentially expressed (Differentially Expressed Genes(DEGs): FC > 2, P value <0.05) and differentially methylated (Differentially Methylated Positions(DMPs): FC > 1.2, P value <0.05). Through this approach, we identified 300 genes common to both datasets.

### 2.4 Cartesian product

To overcome the limitations of HDLSS, integrating the gene expression dataset with the DNA methylation dataset is crucial. It provides a comprehensive approach to understanding gene function and regulatory mechanisms when targeting the same gene.

**FIGURE 3:**
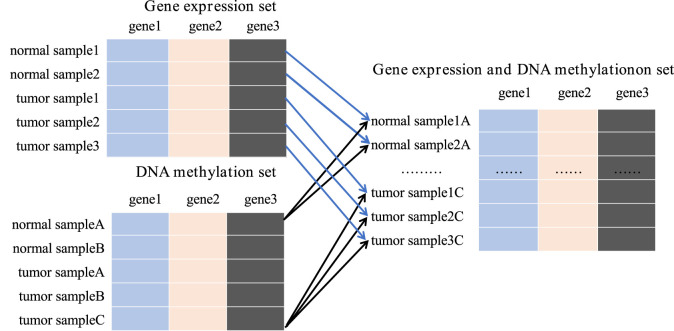
The process of Cartesian product.

To achieve this, we integrated all available gene expression and DNA methylation data from tumor samples and normal samples using the Cartesian product into one comprehensive dataset. New tumor samples were constructed by amalgamating gene expression and methylation data labeled as tumor, while new normal samples were similarly compiled from corresponding normal data. For instance, the gene expression dataset contains 167 samples, including 52 tumor samples and 115 normal samples. The DNA methylation dataset includes 132 samples, with 66 tumor samples and 66 normal samples. Upon integration, we obtained 3,432 tumor samples and 7,590 normal samples, totaling 11,022 samples in the new data set. This substantial dataset scale provides a robust foundation for subsequent studies (Figure 3).

### 2.5 Adjacency matrix construction

To apply omics data to a multi-level attention graph neural network (MLA-GNN), it is necessary to create an adjacency matrix that reflects the relationships between genes. This process often involves using a bioinformatics technique known as weighted gene co-expression network analysis(WGCNA). WGCNA examines pairwise correlations between variables, typically genes, to construct a graph network that represents the strength and direction of associations among genes based on their expression profiles (Pei et al., 2017). Assuming that there are N patient samples, each sample contains the expression values of K genes, then the expression of each gene can be expressed as a N dimensional vector. For any two genes (nodes) i and j , the WGCNA between them is calculated as follows:

Extraction of gene expression vectors: extract the expression vectors of gene i and j from the training data, denoted as $x_{i\;}$ and $x_{j}$ , respectively. These two vectors are N dimension, with each element representing the expression level of the gene in a patient sample.

Calculating correlation: use Pearson correlation coefficient to calculate the correlation between $x_{i}$ and $x_{j}$ .

The specific conversion is shown in Formula 1, for the training data $X^{K\times N}$ (where N denotes the number of patients in the training set and K denotes the number of corresponding genes in each patient), the expression of each node (gene) is characterized by K -dimensional vectors derived from N samples. For any two nodes $x_{i}$ and $x_{j}\in R^{N}$ , the correlation $C_{ij}$ between them is calculated as follows:

$$C_{ij}=\frac{1}{2^{\alpha }}{\left(1+\frac{\sum_{n=1}^{N}\left(x_{i,n}-{\;\bar{x}}_{j}\right)\left(x_{j,n}-{\bar{x}}_{j}\right)}{\sqrt{\sum_{n=1}^{N}{\left(x_{i,n}-{\bar{x}}_{i}\right)}^{2}}\sqrt{{\sum_{n=1}^{N}\left(x_{j,n}-{\bar{x}}_{j}\right)}^{2}}}\right)}^{\alpha }$$ (1)

where ${\;\bar{x}}_{i}$ and ${\bar{x}}_{j}$ are the average values of $x_{i}$ and $x_{j}$ , respectively. The adjacency matrix is obtained by applying a power transformation to the correlation matrix computed by WGCNA analysis, using a soft threshold α . This soft threshold α is automatically determined by the ‘pickSoftThreshold’ function in the WGCNA package.

When constructing the adjacency matrix, incorporating all relevant information into the graph structure can lead to information redundancy and include a significant amount of redundant data and noise. If these features are used directly for training, they may negatively impact the model’s generalization performance. The specific conversion is shown in Formula 2, a soft queer value $adj_{thersh}$ is applied to the adjacency matrix to generate the edge matrix A , and the continuous value C in the adjacency matrix are then processed using the following formula to obtain the matrix A :

$$A_{ij}=\left\{\begin{array}{l} 1,C_{ij}>adj_{thersh}\\ 0,otherwise \end{array}\right.$$ (2)

where the hyperparameter $adj_{thersh\;}$ is optimized by an automatic machine learning algorithm (Xing et al., 2022). The edge matrix A is calculated based on all the training data. Using the edge matrix A , it is possible to transform the gene expression data of each patient into an intuitive gene co-expression map.

### 2.6 MLA-GNN construction

**FIGURE 4:**
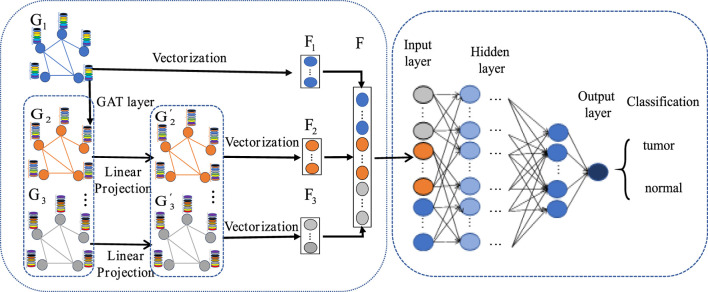
MLA-GNN model.

The MLA-GNN construction centers on transforming each patient’s gene expression data into a structural graph. This graph is then integrated into a network where the adjacency matrix is derived from WGCNA analysis. A crucial element is the stacked Graph Attention Network (GAT), which employs self-attention to handle graph data. This mechanism ensures that node features represent a weighted blend of neighboring nodes and their own characteristics (Velikovi et al., 2017; Qiu et al., 2024), with weights determined by node connectivity and features (Figure 4).

Regarding the construction of the structural map, stacking two GAT layers yields three important outputs from the graph convolution network: the original gene expression matrix ${\;G}_{1}$ , a new gene expression matrix $G_{2}$ that is obtained by weighting and combining through the GAT layers, and the weighted expression matrix $G_{3}$ that results from an additional convolution with the GAT layers. The original gene expression matrix reflects the differences in genes between patients and normal individuals; while the latter two matrices further combine the correlations between genes and assign them corresponding weighted values, resulting in new gene-sample expression relationships.

To ensure consistency between the original gene expression matrix and the newly weighted gene expression matrix, we employ linear projection via fully connected layers. This approach generates high-level graph features $G_{2}^{\prime }\text{ and }G_{3}^{\prime }$ that maintain uniform weighting across datasets. By addressing potential weight imbalances introduced by the GAT layer, each dataset contributes equally to the final outcome.

Due to the impressive outcomes demonstrated by full gradient saliency (FGS) in graph neural networks (Srinivas and Fleuret, 2019), we apply this full gradient saliency mechanism to elucidate the MLA-GNN model and assess node significance. Typically, these nodes represent gene expression within the MLA-GNN framework, with their importance potentially impacting both locally and globally.

## 3 Results

In this paper, we propose three hypotheses: first, predicting LIHC using multi-omics dataset lead to higher accuracy than using single-omics dataset; second, a biometric feature selection method (Differentially Expressed Gene(DEG) + Differentially Methylated Position(DMP)) outperforms standard dimensionality reduction algorithms in LIHC prediction; and third, the MLA-GNN classifier outperforms traditional classifiers in LIHC prediction.

To test these hypotheses, we divided our experiment into three parts: (I) comparing prediction model accuracies using different dataset types; (II) performing dimensionality reduction with MI, T-SNE, and biometric feature selection methods; and (III) comparing the performance of traditional classifiers (DNN, SVM, and NB) with the MLA-GNN model during prediction.

In this paper, the proposed MLA-GNN model input layer is composed of integrated GE and DNA methylation data. For fair evaluation, we compare the developed MLA-GNN method with conventional machine learning-based (ML) and deep learning-based (DL) classifiers. The classifiers discussed are: Deep Neural Networks (DNN), Support Vector Machines (SVM), and Naive Bayes (Table 2).

**TABLE 2:** Parameter configuration.

Methods	Parameter setup
DNN	learning rate = 0.02, dropout = 0.6
SVM	kernel = “linear”, C = 1, probability = True
NB	shuffle = True, random_state = 42
MLA-GNN	learning rate = 0.005, dropout = 0.4

### 3.1 Comparison between single-omics dataset and multi-omics dataset

In processing the multi-omics dataset, we follow procedures similar to those of single-omics dataset, selecting the top 300 significant features for each type of omics data. By utilizing the MI and T-SNE algorithms, we extracted dimensions that aligned with the study’s feature selection criteria. To assess prediction performance, we compared the conventional ML-based learning models such as SVM, RF and DL-based model such as DNN. demonstrates that the feature selection methods proposed in this paper (DEG and DMP) outperform MI or T-SNE alone across multiple datasets (Table 3).

**TABLE 3:** The AUROC results of different prediction algorithms.

	Gene expression			DNA methylation			Gene expression and DNA methylation		
	MI	T-SNE	DEG	MI	T-SNE	DMP	MI	T-SNE	DEG + DMP
SVM	0.94	0.90	0.95	0.92	0.90	0.96	0.95	0.94	**0.97**
DNN	0.92	0.92	0.95	0.91	0.90	0.93	0.93	0.97	**0.98**
RF	0.92	0.93	0.96	0.88	0.91	0.94	0.91	0.96	**0.99**

Bold values indicate the distribution of maximum values for the feature selection methods compared.

Specifically, applying DEG feature selection to gene expression data and using it as input for the SVM prediction model yielded an AUC of 0.95 for LIHC. This accuracy surpassed that achieved by the T-SNE method by 0.05 and the MI method by 0.01. Similarly, DNA methylation data post-DMP feature selection also achieved a high AUC of 0.96.

Additionally, we conducted a comparative analysis of single-omics dataset and multi-omics dataset, evaluating nine combinations that included various feature extraction methods and data types.visualizes AUROC values across different datasets and feature selection methods, emphasizing the robustness and effectiveness of DEG and DMP feature selection methods across diverse classifiers (SI Appendix, Supplementary Figure S1).

Comparing AUC values across various classifiers using different feature selection methods in both single-omics dataset and multi-omics datasets (Table 3, SI Appendix, Supplementary Figure S1), the combining of DEG + DMP feature selection proved more effective in integrating GE and DNA methylation datasets for hepatocellular carcinoma classification.

### 3.2 The impact of different classifiers on performance

In comparing single-omics dataset and multi-omics dataset, we identified the DEG + DMP feature selection method as particularly effective. To comprehensively assess model performance, we examined various classifiers under this feature selection approach and detailed our findings (Table 4).

**TABLE 4:** Average test results based on learning algorithms.

	Gene expression and DNA methylation			
	Accuracy	Precision	Recall	F1-score
DNN	0.98	0.97	0.95	0.98
NB	0.95	0.94	0.92	0.91
**MLA-GNN**	**0.98**	0.98	**0.98**	**0.98**
SVM	0.95	**1.00**	0.94	0.96

Bold values represent the distribution of maximum values for the classifiers compared.

Illustrate that MLA-GNN excels across several evaluation metrics (SI Appendix, Supplementary Figure S2). While slightly trailing SVM in precision, MLA-GNN significantly outperforms DNN in metrics like Accuracy and Recall, and remains competitive with SVM and NB. Notably, MLA-GNN uses its unique self-attention mechanism to reveal intrinsic gene relationships, which further enhances classification performance. The experimental outcomes unequivocally demonstrate MLA-GNN’s efficiency and efficacy in classification tasks, highlighted by metrics such as average accuracy, F1-score, and AUC value, thereby underscoring its robust applicability in relevant domains.

## 4 Discussion

During the key gene screening process, we utilized feature selection in conjunction with MLA-GNN as a classifier to identify genes pivotal to specific biological processes or disease states.

Initially, we employed a feature selection algorithm to filter genes. This step aims to identify a subset of genes that significantly contribute to the classification task, thereby reducing redundancy and enhancing both classifier performance and interpretability.

Subsequently, we developed a graph network model of gene expression where genes represent nodes and their interactions or regulatory relationships represent edges. Using the graph neural network, we captured the intricate network structure’s information and learned node feature representations effectively. We evaluated each gene’s importance by utilizing the gene representations learned through MLA-GNN and their performance in the classification task.

Ultimately, based on the feature selection results, we identified key genes that make substantial contributions to the classification task. These key genes will serve as focal points in subsequent biological experiments or clinical studies.

To maintain clarity and coherence in our discussion, we have separated the key gene screening results from the subsequent empirical analyses into different sections. In the empirical analysis section, we will present the performance and validation outcomes of these key genes in clinical and drug trials.

### 4.1 Screening key genes

**FIGURE 5:**
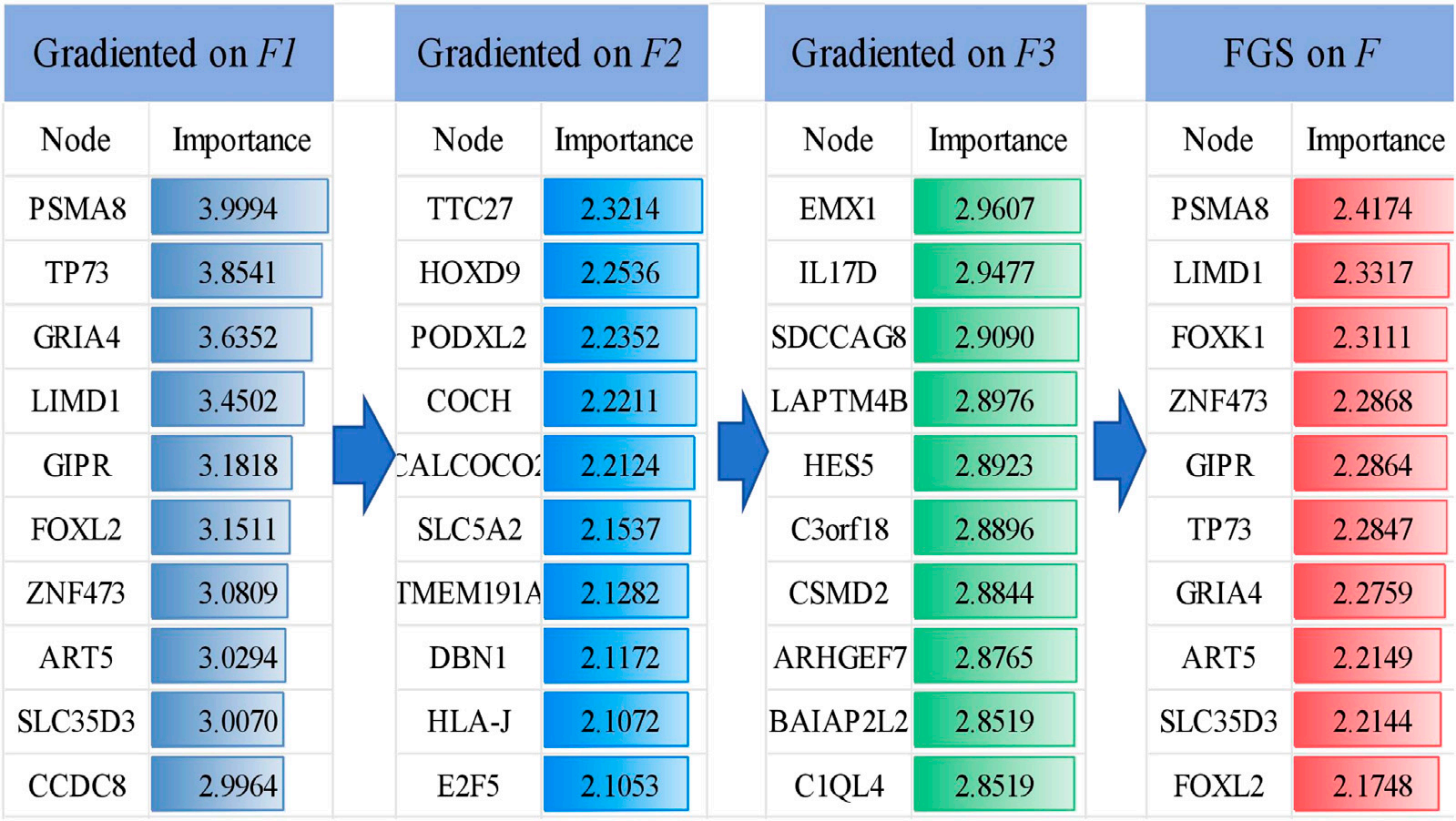
Visualization of the TOP 10 important genes and their gradient-based saliency scores in each layer ( $F_{1},{\;F}_{2}$ and $F_{3}$ ) and after fusion (FGS on F ).

The FGS saliency algorithm was used to identify critical nodes and perform detailed analysis at deeper layers (Figure 5). Illustrates this process, highlighting PSMA and TP73 as pivotal biomarkers in liver cancer at the $F_{1}$ level. Subsequently, the analysis identified the transcription factor EMX1, which promotes hepatocellular carcinoma metastasis through the EMX1-EGFR-ERK axis in the $F_{3}$ tier (Wen et al., 2023).

### 4.2 Key genes empirical analysis

To evaluate the potential of these 10 genes in identifying LIHC, we conducted a gene analysis. After reviewing the literature, we found that the PSMA8 gene is associated with tumor cell invasion and metastasion (Li et al., 2024). The upregulation of the LIMD1 gene may be due to hypomethylation of the promoter through downregulation of DNMT1 expression (Pal et al., 2021). Inhibition of the Akt/mTOR pathway by FOXK1 reduces cell viability and glycolysis in hepatocellular carcinoma cells (Cui et al., 2018). ZNF473 has been reported as a diagnostic marker in various cancers (Bulanenkova et al., 2022; Lai et al., 2023). The expression of TP73 is specific to the cancer cell line and not to the neighboring normal liver tissue (Yao et al., 2019). GRIA4 hypermethylation is significantly increased in primary tumors and liver metastases (Lukacova et al., 2023). GIPR is highly prevalent (approaching 100%) in both functional and non-functional pancreatic tumors (Reubi et al., 2020). GIPR produces effects in non-alcoholic fatty liver disease and liver fat (Yao et al., 2019). ART5 has significant prognostic value in colorectal cancer. SLC35D3 is highly expressed in colorectal cancer (CRC) tissue (Geng et al., 2024). FOXL2 can significantly induce apoptosis in cancer cells (Han et al., 2019).

Selected sets of genes underwent analysis using the DAVID database to determine their biological significance (Dennis et al., 2003), which is detailed in Table 5. The following Gene Ontology (GO) terms were identified:

**TABLE 5:** GO analysis of selected genes.

Category	Term (GO)	P value	Genes
GOTERM_MF_DIRECT	0000978	0. 010	ZNF473, FOXK1, FOXL2, TP73
GOTERM_MF_DIRECT	0001228	0. 017	ZNF473, FOXL2, TP73
GOTERM_BP_DIRECT	0045892	0. 021	FOXK1, FOXL2, LIMD1
GOTERM_BP_DIRECT	0006357	0. 023	ZNF473, FOXK1, FOXL2, TP73
GOTERM_MF_DIRECT	0003700	0. 025	FOXK1, FOXL2, TP73
GOTERM_BP_DIRECT	0045893	0. 033	FOXK1, FOXL2, TP73

“GO: 0000978∼RNA polymerase II core promoter proximal region sequence-specific DNA binding”in RNA polymerase II-mediated transcription initiation (Martin et al., 2020); “GO: 0001228∼transcriptional activator activity” involved in promoting or repressing the expression of tumor-associated genes (Xiao et al., 2020); “GO: 0045892∼negative regulation of transcription, DNA-templated” associated with gene silencing or expression inhibition in cancer; “GO: 0006357∼regulation of transcription from RNA polymerase II promoter “describes the process of DNA template based transcription; “GO: 0003700∼transcription factor activity, sequence-specific DNA binding” is significantly associated with the expression of β-globin by cancer cells (Zheng et al., 2017); “GO: 0045893∼positive regulation of transcription, DNA-templated “plays a role in hepatitis B (Zhang et al., 2020).

**FIGURE 6:**
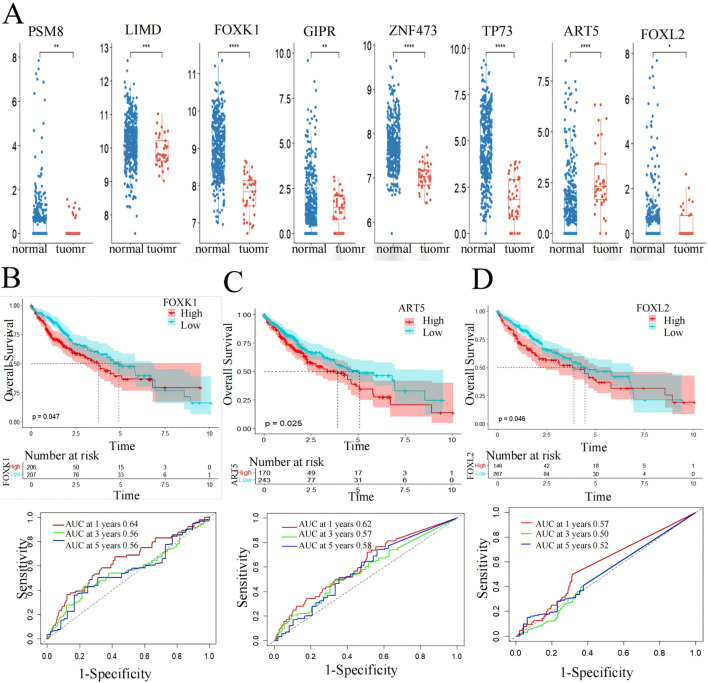
Screening markers of liver cancer by 10 genes. **(A)** expression levels in LIHC patients from the TCGA cohort. *p < 0.05, **p < 0.01, ***p < 0.001. **(B)** FOXK1, **(C)** FOXL2 and **(D)** ART5 have significant effects on the prognosis of overall survival of LIHC (p < 0.05) and AUC values for predicting the 1-,3- and 5-year survival rates of patients with LIHC.

Box plot and Kaplan-Meier survival curve analysis were conducted on 10 genes identified in 424 LIHC patients using the TCGA database (Figure 6). These genes were initially screened through a model that integrates gene expression and DNA methylation datasets from the GEO and hepatocellular carcinoma data. The validation results of these genes were displayed using the TCGA gene expression database, showing significant differences (p < 0.05) in eight genes between the patient group and the normal group, where the gene expression in the patient group was higher than that in the normal group (Figure 6A). Meanwhile, the core genes screened in this paper were based on DNA hypermethylation and upregulated expression, suggesting that hypermethylation may promote their high expression.

Subsequently, the eight genes showing significant differences underwent Kaplan-Meier survival curve analysis and ROC curve analysis for prognostic evaluation (Figures 6B–D). Among these, FOXK1 and FOXL2 demonstrated statistically significant differences (p < 0.05) in survival analysis. ART5 also showed promising results as indicated by the RiskScore predicting favorable 1-, 3-, and 5-year AUC values, except for FOXL2, which showed less ideal results (3-year: 0.5, 5-year: 0.52). These findings suggest the potential utility of FOXK1, FOXL2, and ART5 expression levels as biomarkers for LIHC, highlighting their role in prognostic assessment and clinical implications.

Utilizing functional enrichment analysis from the DAVID database and Kaplan-Meier survival curve analysis from the TCGA database, we identified significant enrichment of FOXK1 and FOXL2 in various biological pathways, particularly those linked to LIHC. The Kaplan-Meier analysis revealed a strong association between these genes and patient survival, with P values below 0.05, underscoring their potential as prognostic indicators in liver cancer.

Furthermore, box plot validation from the TCGA database confirmed that FOXL2 expression was markedly elevated in LIHC patients compared to healthy individuals. This observation bolsters the evidence for FOXL2’s role in LIHC progression. Since FOXK1 is already established as an LIHC biomarker, our findings imply that FOXL2 could also be a valuable marker for LIHC, aiding in both diagnosis and prognosis.

### 4.3 Correlation analysis of drug sensitivity

FOXL2’s potential involvement in drug resistance prompted us to obtain gene expression and drug sensitivity data from the CellMiner database (Luna et al., 2021). We rigorously filtered out drugs not tested in clinical trials or approved by the FDA. Using the cor. Test function in R, we calculated correlation coefficients between FOXL2 expression and drug sensitivity. Based on the R-values, we identified the top 9 drugs most strongly correlated with FOXL2 expression.

**FIGURE 7:**
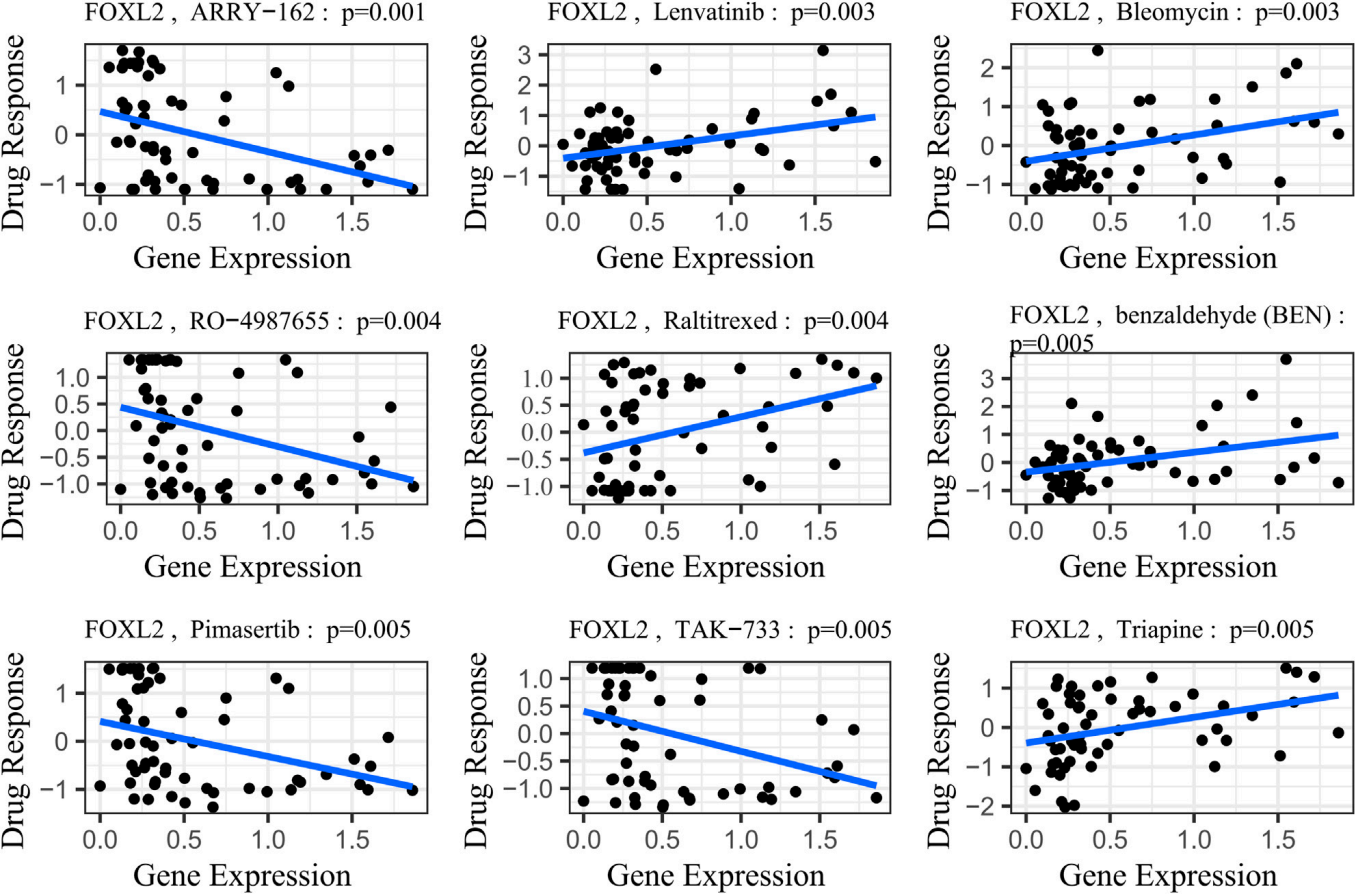
Gene-drug sensitivity analysis based on the CellMiner database; the top 9 drugs with high correlation with gene expression in inflammation-related prognostic models were screened. Related prognostic models were screened.

The study results demonstrated a significant positive correlation between FOXL2 gene expression and drug sensitivity (Figure 7, SI Appendix; Supplementary Table S1). Specifically, FOXL2 exhibited statistically significant associations with several drugs: Lenvatinib (cor = 0.38, p = 0.003), benzaldehyde (cor = 0.442, p = 0.001), Bleomycin (cor = 0.37, p = 0.003), Raltitrexed (cor = 0.36, p = 0.004), and Triapine (cor = 0.36, p = 0.005). Notably, Lenvatinib, a multikinase inhibitor, is used to treat various cancers, including liver and thyroid cancers (Suyama and Iwase, 2018). Bleomycin is an antitumor antibiotic that inhibits cell growth by damaging DNA (Wang et al., 2013). Triapine is an iron-binding ligand and anticancer drug acid (Ratner et al., 2016).

To illustrate this trend more clearly, we stratified gene expression into high-risk and low-risk groups based on the median. We then extracted the IC50 values associated with these groups and presented them in a box plot (SI Appendix, Supplementary Figure S3). Following a Wilcoxon rank sum test, we observed statistically significant differences in drug sensitivities associated with FOXL2 gene expression, except for Bleomycin (*p*-value = 0.066), for which the difference was not significant. This finding underscores the important role of the FOXL2 gene in influencing responses to specific classes of drugs.

FOXL2 expression exhibited significant negative correlations with drug sensitivities, notably with ARRY-162 (cor = –0.40, p = 0.001), RO–4987655 (cor = -0.37, p = 0.001), Pimasertib (cor = –0.36, p = 0.001), and TAK-733 (cor = –0.36, p = 0.001) (Figure 7, SI Appendix; Supplementary Table S1). ARRY–162 (Binimetinib) is used for treating melanoma and other cancers (Lee et al., 2009). Ulixertinib is used to treat non-small cell lung cancer (Sullivan et al., 2018). Vinorelbine, a chemotherapy agent, is prescribed for non-small cell lung and breast cancer (Galano et al., 2011). After performing the Wilcoxon rank sum test, we observed that all differences in drug sensitivities were statistically significant (SI Appendix, Supplementary Figure S3).

In our study, we identified Lenvatinib, a multikinase inhibitor with proven clinical efficacy in HCC, as a key therapeutic target by analyzing drug sensitivity data. Our analysis revealed a significant correlation between FOXL2 gene expression and Lenvatinib sensitivity, suggesting FOXL2’s potential as a predictive biomarker for drug response in HCC patients.

Supported by clinical and preclinical evidence, Lenvatinib targets VEGF and FGFR pathways critical for HCC progression, as demonstrated by Suyama and Iwase (2018) and confirmed by the phase III REFLECT trial (Watanabe and Koyama, 2019; Kim et al., 2020). This correlation between FOXL2 expression and Lenvatinib sensitivity highlights the importance of FOXL2 in HCC. Our findings indicate that FOXL2 may serve as a HCC biomarker and a predictor of Lenvatinib sensitivity, warranting further investigation into its role in HCC and its clinical application in treatment decision-making.

### 4.4 Molecular docking of FOXL2 gene with lenvatinib

**FIGURE 8:**
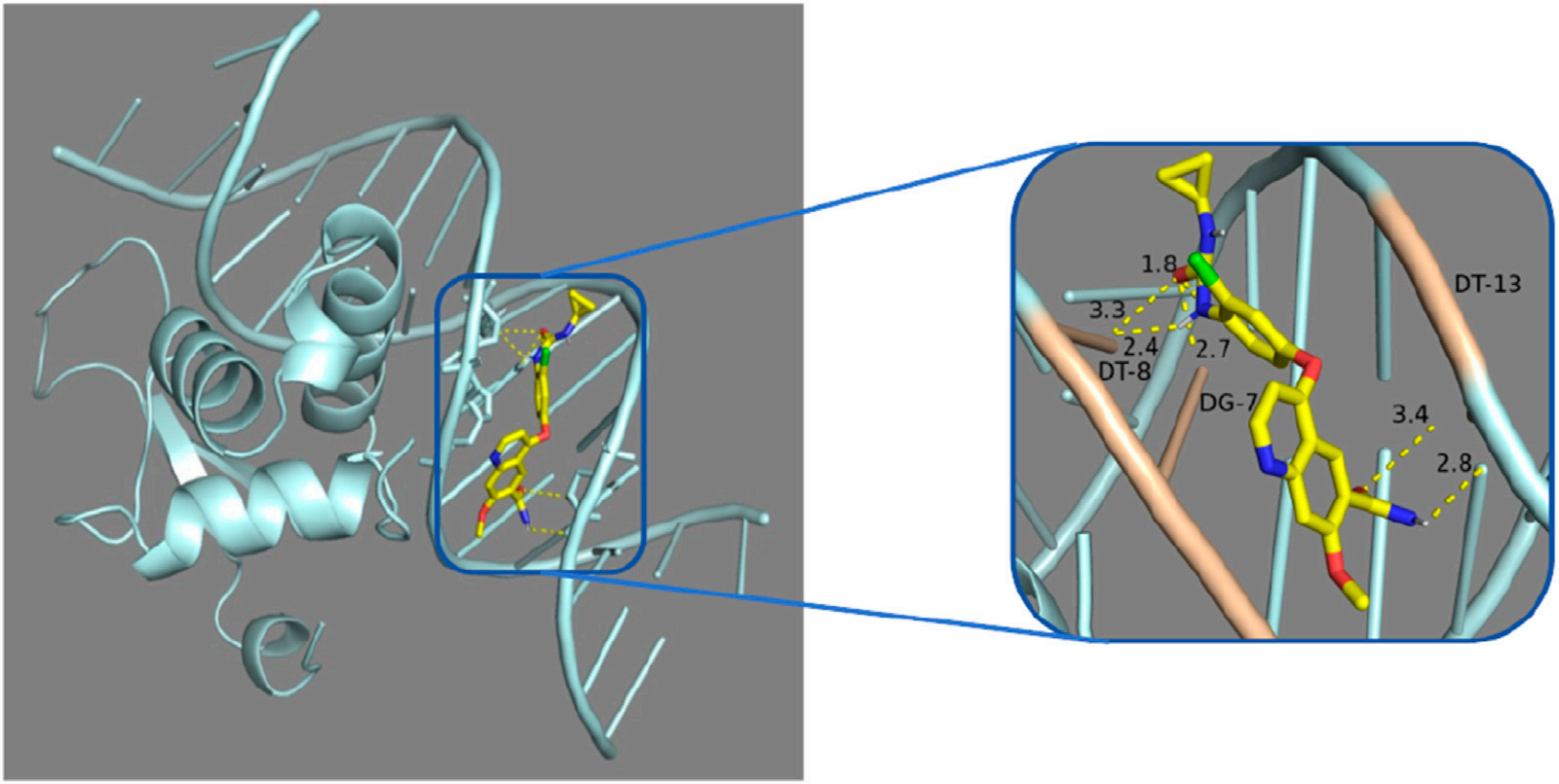
Molecular docking analysis: Lenvatinib was docked with 7VOU.

In our drug sensitivity analysis, we identified a significant positive correlation between FOXL2 and Lenvatinib, suggesting that FOXL2 may serve as a potential biomarker for liver cancer therapy. To further investigate this correlation, we conducted molecular docking experiments using Vina software to assess the potential of compounds binding to FOXL2. According to literature and industry standards, molecular docking results with binding energies below −7 kcal/mol are considered as candidate drugs (Apaer et al., 2024), as such binding affinity is typically associated with the efficacy and potency of drugs. In our experiments, Lenvatinib exhibited a binding energy of −8.5 kcal/mol, which is well below the industry threshold, indicating a very strong interaction between Lenvatinib and FOXL2 (Table 6; Figure 8). These experimental results not only support the notion that FOXL2 is a potential biomarker for liver cancer treatment but also provide significant molecular evidence for the development of new therapeutic strategies.

**TABLE 6:** Chemical drugs with therapeutic potential.

Target name	Protein names	PDB ID	Compound name	Binding energy (kj/mol)
FOXL2	Forkhead box protein L2	7VOU	Lenvatinib	−8.5

In our study, through molecular docking technology, we found that the key gene FOXL2 can effectively bind to the compounds, Lenvatinib. This discovery is of great significance for understanding the mechanism of action of these compounds in immune regulation, especially for diseases such as Liver Hepatocellular Carcinoma (LIHC). Lenvatinib is an established and promising drug for the treatment of advanced hepatocellular carcinoma (Catalano et al., 2021). Its mechanism of treatment for hepatocellular carcinoma has shown significant results from preclinical studies to anticancer therapy (Zhao et al., 2020).

## 5 Conclusion

In this study, we applied the MLA-GNN model to analyze LIHC and identified key biomarkers using integrated multi-omics data. Our comparison of various feature selection methods and models revealed the superior performance of the method proposed in this study. Its notable advantage lies in integrating multi-omics data without blind dimensionality reduction, thereby allowing for the selection of biologically significant features. Biological correlation analysis and literature validation further supported the potential of these genes as LIHC biomarkers. Despite our exhaustive bioinformatics analysis, this study has limitations. For example, given that WGCNA emphasizes the importance of positive correlation in constructing gene co-expression networks, and that the ReLU activation function effectively avoids the problem of gradient disappearance by passing only positive gradients and ignores genes negatively correlated with the target category, we selected only upregulated genes in DNA methylation and gene expression. Moving forward, we aim to further explore this method’s application with similar histological data and refine our research strategy accordingly.

## Data Availability

The datasets presented in this study can be found in online repositories. The names of the repository/repositories and accession number(s) can be found in the article/Supplementary Material.
