# Supplementary material for: Integration of gene expression and DNA methylation data using MLA-GNN for liver cancer biomarker mining
Source: Front Genet. 2024 Dec 23;15:1513938. doi: 10.3389/fgene.2024.1513938 (PMC11701154; doi:10.3389/fgene.2024.1513938)
Supplement: Supplementary file 1 [file DataSheet1.docx]

Supplementary Material

# **Supplementary Figures and Tables**

## Supplementary **Tables**

**Table S1.** Correlation analysis between FOXL2 gene and drugs.

| Gene | Drug | cor | pvalue |
| --- | --- | --- | --- |
| FOXL2 | ARRY-162 | -0.40 | 0.001 |
| FOXL2 | Lenvatinib | 0.38 | 0.003 |
| FOXL2 | Bleomycin | 0.37 | 0.003 |
| FOXL2 | RO-4987655 | -0.37 | 0.004 |
| FOXL2 | Raltitrexed | 0.36 | 0.004 |
| FOXL2 | benzaldehyde (BEN) | 0.36 | 0.005 |
| FOXL2 | Pimasertib | -0.36 | 0.005 |
| FOXL2 | TAK-733 | -0.36 | 0.005 |
| FOXL2 | Triapine | 0.36 | 0.005 |

## Supplementary Figures

**Figure S1.** Average AUC of all comparisons.

**Figure S2.** Comparison of experimental results of GNN with other methods.


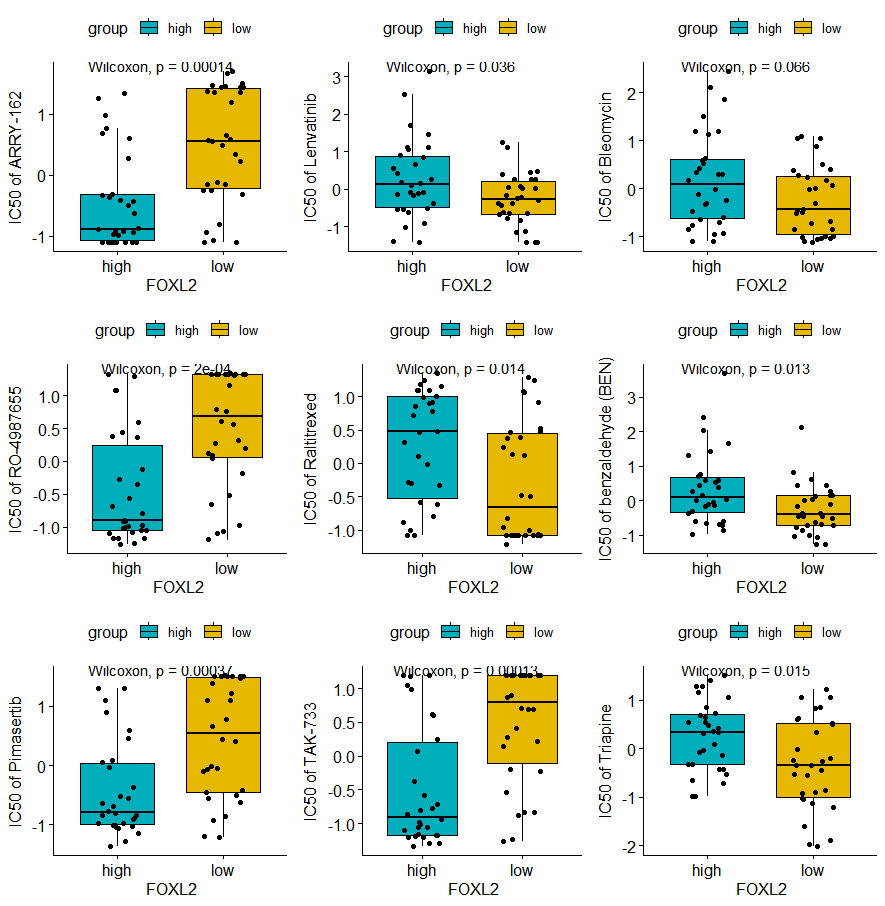


**Figure S3.** Box plots showing the top 9 drugs in samples with high and low FOXL2 expression.
